# Supplementary material for: Hemolymph Ecdysteroid Titer Affects Maternal mRNAs during Bombyx mori Oogenesis
Source: Insects. 2021 Oct 27;12(11):969. doi: 10.3390/insects12110969 (PMC8622876; doi:10.3390/insects12110969)
Supplement: Supplementary file 1 [file insects-12-00969-s001.zip › insects-1388458-supplementary.pdf]

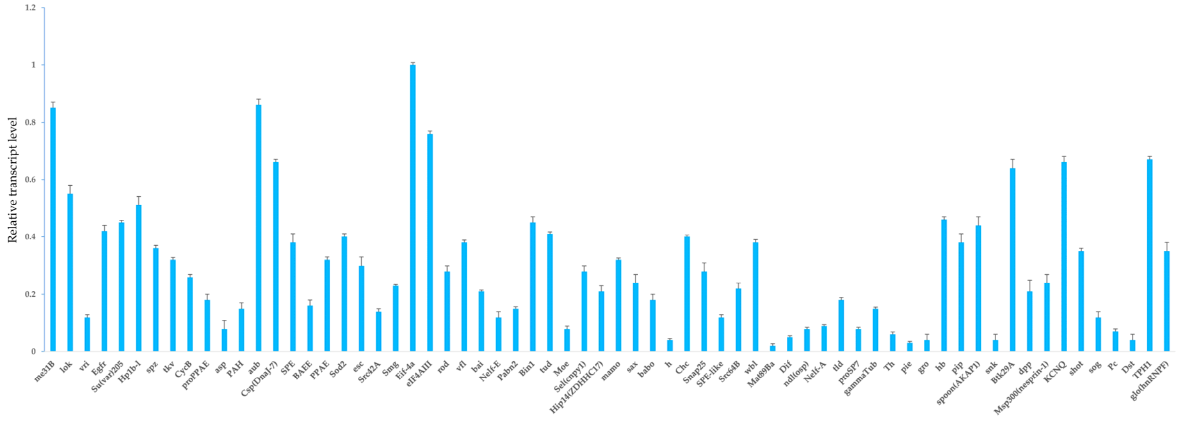

Figure S1. Expression patterns of maternal genes in the ovary of the wandering silkworm by reverse-transcription quantitative PCR (RT-qPCR). TIF-4A was used as the internal control, and the relative quantity of TIF-4A was set to 1. Bars represent the standard deviation.

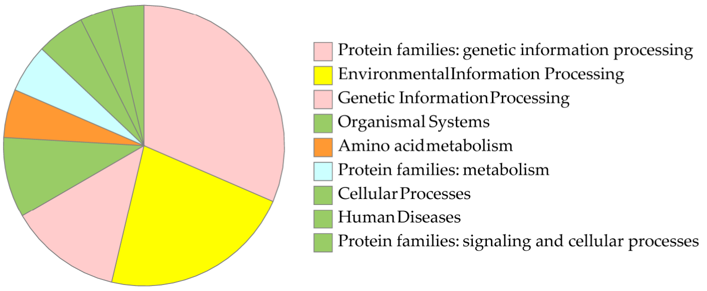

Figure S2. Kyoto Encyclopedia of Genes and Genomes KEGG analysis of maternal genes.

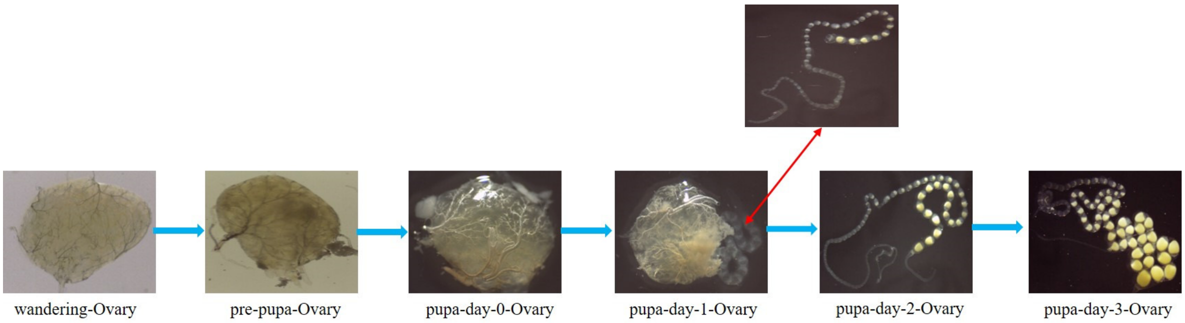

Figure S3. View of the ovary or ovariole of the silkworm from the wandering larvae to day-3 pupae.

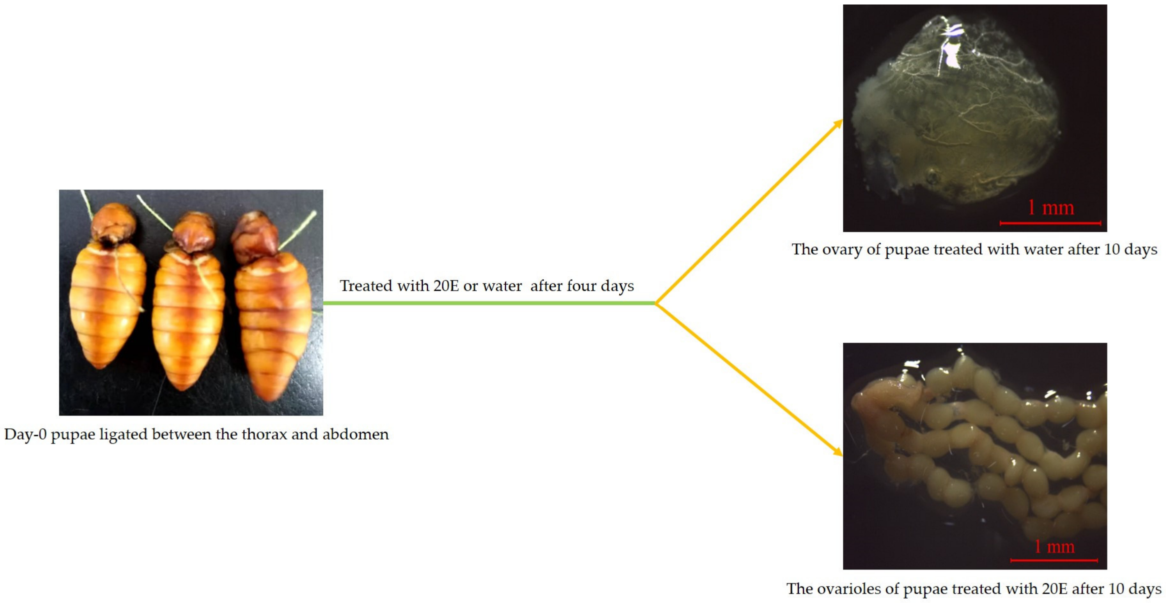

Figure S4. View of the 20E-depleted abdomens and the ovary or ovariole of the 20E-depleted abdomens injected with 5 μL of a 20E (10.0 μg solution or 5 μL of sterile distilled water.
